# Year in review 2013: *Critical Care* – out-of-hospital cardiac arrest, traumatic injury, and other emergency care conditions

**DOI:** 10.1186/s13054-014-0593-y

**Published:** 2014-10-29

**Authors:** Scott A Goldberg, Bryan Kharbanda, Paul E Pepe

**Affiliations:** Department of Emergency Medicine, Brigham and Women’s Hospital, 75 Francis Street, Neville House, Boston, MA 02115 USA

## Abstract

In this review, we discuss articles published in 2013 contributing to the existing literature on the management of out-of-hospital cardiac arrest and the evaluation and management of several other emergency conditions, including traumatic injury. The utility of intravenous medications, including epinephrine and amiodarone, in the management of cardiac arrest is questioned, as are cardiac arrest termination-of-resuscitation rules. Articles discussing mode of transportation in trauma are evaluated, and novel strategies for outcome prediction in traumatic injury are proposed. Diagnostic strategies, including computerized tomography scan for the diagnosis of smoke inhalation injury and serum biomarkers for the diagnosis of post-cardiac arrest syndrome and acute aortic dissection, are also explored. Although many of the articles discussed raise more questions than they answer, they nevertheless provide ample opportunity for further investigation.

## Introduction

Several articles published in 2013 enhanced existing literature regarding the management of out-of-hospital cardiac arrest (OHCA) and the evaluation and management of several other emergency conditions, including traumatic injury. Articles took issue with the utility of established management protocols and challenged existing algorithms. The utility of intravenous (IV) medications in the management of cardiac arrest was examined, with specific attention to epinephrine and anti-arrhythmics, highlighting a paucity of clear evidence supporting their use. The appropriate timing for termination of resuscitative efforts in cardiac arrest was likewise called into question, drawing attention to potential shortcomings of existing guidelines. Mode of transportation to the hospital in trauma and its effect on outcomes were evaluated, suggesting a potential benefit of helicopter transport but providing no definite answer as to the most efficacious transport modality. Novel strategies for predicting outcomes in traumatic injury by using inexpensive, readily available indices such as the shock index (SI) and base deficit (BD) were proposed. Diagnostic strategies, including computerized tomography (CT) scan for the diagnosis of smoke inhalation injury (SII) and serum biomarkers for the diagnosis of post-cardiac arrest syndrome and acute aortic dissection, were also explored. Here, we review these emergency care studies and comment on their clinical application.

## Utilization of intravenous medications in cardiac arrest

Despite recent advances in medical technology and therapeutics, the morbidity and mortality of cardiac arrest remain high [1]. According to contemporary reports, the estimated mortality rate for OHCA is still in the range of 92% to 96% [1,2]. In recent years, the focus of resuscitative efforts in cardiac arrest has moved toward continuous chest compressions and the quality of basic life support maneuvers [3-5]. Nevertheless, the use of IV medications remains an integral part of advanced life support (ALS) algorithms [6,7]. However, the safety and efficacy of such medications are increasingly questioned [8,9]. Last year saw the publication of several articles related to cardiac arrest that raised additional concerns regarding the utility of IV medication administration in ALS algorithms.

### Anti-arrhythmic drugs for out-of-hospital cardiac arrest

Despite a paucity of evidence of long-term benefit, anti-arrhythmic agents are recommended as a part of standard resuscitation algorithms for persistent ventricular arrhythmias without palpable pulses following attempted cardioversion [6,7]. In addition, new agents have been used for ventricular fibrillation (VF) and ventricular tachycardia (VT) in the years following the most recent iterations of international guidelines. Therefore, Huang and colleagues [10] undertook a systematic literature review and meta-analysis evaluating studies of cardiac arrest in patients over age 18 in which an anti-arrhythmic was used, regardless of the presenting cardiac rhythm. The final analysis included 14 studies of varying quality. The populations studied were heterogeneous in terms of in-hospital cardiac arrest and OHCA as well as initial rhythm.

Pooled results from evaluated randomized trials did not demonstrate any significant improvement in survival to discharge for any agent, including amiodarone (risk ratio (RR) = 0.82, 95% confidence interval (CI) = 0.54 to 1.24), magnesium (RR = 1.07, 95% CI = 0.62 to 1.86), or lidocaine (RR = 2.26, 95% CI = 0.93 to 5.52). Although there was no long-term advantage, lidocaine was associated with improved survival to admission whereas amiodarone and magnesium were not. Nevertheless, pooled analysis demonstrated no significant difference in survival to either hospital admission or discharge when amiodarone was compared directly with lidocaine (*P* = 0.28). None of the evaluated studies reported neurologic outcome measures.

In addition to these more traditional anti-arrhythmic agents, newly introduced potassium channel blockers such as nifekalant were analyzed as well. Four observational studies were evaluated, and all demonstrated inferior survival compared with amiodarone. Although these studies did indicate a possible benefit over lidocaine in terms of return of spontaneous circulation (ROSC), they demonstrated no benefit in survival to discharge.

In essence, this meta-analysis provided no distinct evidence for a survival benefit from any anti-arrhythmic medication in the management of cardiac arrest. This work mirrors previously published literature suggesting the limited, if any, benefit of ALS interventions in cardiac arrest [8,11,12]. Although this article reviewed a smaller number of studies than another recent meta-analysis [13], the results are similar. There may indeed be some utility of these drugs in certain populations, but routine use is of questionable utility and further large randomized trials will need to be conducted. Fortunately, one such clinical trial is under way [14].

### Epinephrine for out-of-hospital cardiac arrest

Epinephrine has been standard practice in cardiac arrest management for decades [15,16]. Epinephrine increases coronary perfusion pressure [17] and has shown benefit in animal models for ROSC after cardiac arrest [15]. However, epinephrine may also have deleterious effects, including myocardial dysfunction, decreased microcirculation, and cerebral hypoperfusion [16,18]. Furthermore, although epinephrine has been shown to improve rates of ROSC, there is limited evidence of long-term benefit [19,20]. In a study by Goto and colleagues [21], the utility of epinephrine for OHCA was again examined, with a particular focus on those patients initially presenting with a ‘no shock indicated’ rhythm.

This study design was a retrospective analysis of prospectively collected data from an OHCA registry in a Japanese cohort. In this system, there is no field termination protocol and all patients are transported to hospital. A single dose of epinephrine may be administered by protocol, and additional doses may be provided only after discussion with a physician. The study endpoint was 1-month survival among those receiving epinephrine. Secondary endpoints were prehospital ROSC and 1-month favorable neurologic outcome, defined as a cerebral performance category (CPC) score of 1 or 2. Of the 209,577 patients evaluated, 92.6% had an initial cardiac rhythm in which a defibrillatory shock was not indicated. Survival at 1 month with intact neurologic status was 1.8%. In the subset of patients with an initial rhythm of VF or VT, those receiving epinephrine had significantly worse 1-month neurologic outcomes (7.0% versus 18.6% with CPC score of 1 or 2, *P* <0.0001). Those with ‘no shock advised’ had improved prehospital ROSC with epinephrine (18.7% versus 3.0%, *P* <0.0001) but had similar rates of good 1-month neurologic outcomes (0.59% versus 0.62%, *P* =0.605). However, those patients receiving epinephrine after 10 minutes had worse 1-month neurologic outcomes (odds ratio (OR) 0.51, 95% CI 0.44 to 0.59). In those receiving rapid drug administration, after adjustment for initial rhythm, epinephrine was independently associated with worse 1-month neurologic outcomes (OR 0.71, 95% CI 0.54 to 0.92).

Although ostensibly the results indicate worse outcomes when epinephrine is administered, this retrospective review examines a univariate analysis, not a controlled clinical trial. A patient presenting with VF, for example, should be expected to fare poorly if they do not respond to initial cardiopulmonary resuscitation (CPR) and defibrillation attempts and thus move on to the epinephrine step of the protocol. Likewise, one could presume the same outcome, though less pronounced, in those non-VT/VF patients refractory to initial basic CPR and airway interventions. In addition, this review is a retrospective database analysis from the unique prehospital care system of Japan, and generalizability to other cohorts may not be possible.

This study by Goto and colleagues adds to the controversy surrounding the utility of epinephrine administration in OHCA and mirrors several previously published studies [16,19,20]. Although there is no clear demonstration of improvement in long-term outcomes, epinephrine has still been associated with an increase in ROSC and 1-month survival. With the recent increase in utilization of novel therapies, including induced hypothermia and extracorporeal life support, further studies are necessary to determine a possible benefit of epinephrine in these cohorts as well. Finally, the addition of nitrates, vasopressin, or steroids to epinephrine may have some utility but these are not yet in widespread use for the purpose of evaluation [22,23]. Until further research is conducted and alternative therapies established, the current consensus is that epinephrine should not be abandoned. However, its utility in cardiac arrest must continue to be investigated, and there is growing skepticism as to its benefits. A much-anticipated, double-blind, placebo-controlled trial planned to begin in 2014 in the UK [24] will hopefully provide some definitive conclusions.

## Out-of-hospital resuscitation from cardiac arrest

As previously discussed, OHCA is generally associated with poor outcomes. Continuation of resuscitative efforts in medically futile patients can be associated with increased resource utilization, cost, and potential hazards to providers. Current guidelines [25] support termination of resuscitation (TOR) according to specific validated criteria [26-28]. These criteria include an unwitnessed arrest without bystander CPR, a non-shockable rhythm, and failure of ROSC prior to transport. Yet not all patients meeting TOR criteria ultimately expire. Determining which patients with OHCA are expected to have a meaningful recovery was the focus of two recent articles by Goto and colleagues [29,30].

### Termination of resuscitation for out-of-hospital cardiac arrest

Previously published TOR rules have focused largely on prehospital application. In a 2013 study, Goto and colleagues [29] sought to develop an emergency department TOR rule for use with their Japanese emergency medical services (EMS) system and compare it with previously validated rules. In this EMS system, field termination is not permitted and transport of all patients is mandatory. As such, the TOR rule of Goto and colleagues was adapted for use after arrival to the emergency department.

In this study, Goto and colleagues first developed and then validated a TOR rule for OHCA by using a database of 495,607 patients. The authors identified the three variables with the highest adjusted ORs for 1-month death and poor neurologic outcome and defined these as their criteria for TOR. The criteria were lack of prehospital ROSC (adjusted OR 25.8, 95% CI 24.7 to 26.9), presenting rhythm in which a shock was not indicated (adjusted OR 2.76, 95% CI 2.54 to 3.01), and unwitnessed arrest (adjusted OR 2.18, 95% CI 2.09 to 2.28). In the validation group, 57.3% of the cohort met all three criteria. Specificity for death at 1 month was 0.903 (95% CI 0.894 to 0.911), positive predictive value was 0.993 (95% CI 0.992 to 0.993), and the area under the receiver operator curve (AUC) was 0.874 (95% CI 0.872 to 0.876). The AUC for 1-month unfavorable neurologic outcome was 0.942 (95% CI 0.941 to 0.944). The same group had an AUC for the American Heart Association-recommended TOR rule [25] of 0.880 (95% CI 0.871 to 0.889) for unfavorable neurologic outcomes.

Despite published guidelines, many providers are reluctant to terminate resuscitative efforts [31,32]. In fact, emergency department providers can be expected to carry on resuscitation for a similar period of time regardless of prehospital course [33]. Failure to terminate resuscitation in medically futile patients is associated with significant cost [34,35]. This study by Goto and colleagues supplemented the existing literature by proposing a TOR rule for use in the emergency department on the basis of prehospital parameters [29]. However, the unique configuration of this EMS system requiring transport of all patients regardless of anticipated futility must be considered. Although in this cohort the proposed TOR rule does perform better than previously published rules, the study cohort is unique in both population and protocol, and this new rule must be prospectively validated in other patient populations prior to any widespread adoption.

### Factors associated with good neurologic outcome in patients not achieving prehospital return of spontaneous circulation

In the previously described study [29], 153 patients meeting all three currently recommended TOR criteria [25] survived with favorable neurologic outcome. Although these survivors represented only 0.002% of the population, under the currently recommended TOR rule, resuscitative efforts in these patients, who ultimately achieved good neurologic outcomes, would have been aborted. Goto and colleagues evaluated these OHCA patients who were transported to hospital without ROSC and who ultimately had good 1-month neurologic outcomes.

Using the nationwide database previously described, Goto and colleagues retrospectively examined 398,121 cases of OHCA [30]. Unlike previous studies [1], this study excluded all patients with prehospital ROSC. In this cohort, overall 1-month survival was 1.89%, slightly higher than that of previous studies [1]; 1,957 patients (0.49%) had a CPC score of 1 or 2 at 1 month. Not surprisingly, presentations of VF or VT with no detectable pulse were the strongest predictors of good neurologic outcome (OR 9.37 and 8.50, respectively). Other predictors were any rhythm other than asystole, call to hospital arrival time of less than 24 minutes, witnessed arrest, and age of less than 65 years. When all four of these conditions were met, 1 in 6 patients survived to 1 month with a good neurologic outcome.

This study mirrors previous publications that indicate the combination of VF/VT, younger age, witnessed arrest, and short transport time is a predictor of good neurologic outcomes following OHCA. However, 13.6% of all patients with a CPC score of 1 or 2 at 1 month had transport times of more than 37 minutes. In addition, 22.4% of patients with 1-month good neurologic outcomes had an initial presenting rhythm of asystole. Under many current field TOR protocols, resuscitation of these patients would have been abandoned. These findings highlight the need for further research into the specific factors that predict the absolute futility of resuscitation. Although the percentage of patients meeting TOR criteria and ultimately surviving with good neurologic outcomes is quite small, it represents an important cohort and one that is essential to identify. Further evaluation into cost-benefit and societal implications of premature termination of resuscitative efforts is needed.

## Mode of transport and effects on outcome in trauma

Helicopter EMS (HEMS) has a long history of use despite controversies regarding effectiveness [36]. Whereas some studies suggest an overall survival benefit of HEMS [37], others have failed to demonstrate any survival benefit over ground EMS (GEMS) transport [38,39]. Helicopter transport has both the logistical advantage of rapid transportation over large distances as well as the provision of advanced skills via a specialized care team. However, debate still exists regarding the optimal staffing configuration of air medical teams, and existing air medical services provide varying combinations of nurses, paramedics, and physicians. For example, HEMS responses in Germany are exclusively physician-staffed, whereas US flight teams generally involve a flight nurse crew member with or without physicians. Controlling for provider skill level, the authors of a study published in 2013 sought to identify any outcome benefit of HEMS over GEMS transports in a German patient cohort from an all-physician EMS system [40]. The authors also sought to identify any impact of on-scene interventions on outcome.

In this study, Andruszkow and colleagues [40] performed a retrospective review of a large German trauma registry. Importantly, owing to the retrospective nature of the database, the study groups were not directly comparable, so the authors used prognostic scores to adjust observed mortality rates in the two groups. Of the 13,220 patients included in the analysis, 37.7% were transported by HEMS and 81.3% were transported to a level I trauma center. Those patients transported by HEMS tended to be younger and male and had significantly higher injury severity scores (ISSs) (26.0 versus 23.7, *P* <0.001) than those transported by ground. After a multivariate logistic regression was performed, the OR for mortality in the HEMS group was 0.75 (95% CI 0.636 to 0.832). The HEMS cohort received more aggressive interventions, including intubation (65.7% versus 40.6%), vasopressors (10.4% versus 7.1%), and thoracostomy (9.3% versus 2.7%). Despite an increased incidence of sepsis (8.9% versus 6.6%), multi-organ dysfunction (33.4% versus 25.0%), and a higher overall ISS, standardized mortality ratios for the HEMS cohort were significantly lower (0.678 versus 0.825, *P* = 0.001). As previous studies have demonstrated a survival advantage of HEMS transport to a level I trauma center, the authors performed a subgroup analysis of only those patients transported to level I trauma centers demonstrating a persistently lower standardized mortality ratio for the HEMS cohort (0.647 versus 0.815, *P* =0.002).

This study contributes to the existing debate as to the potential benefit of air transport over ground. Unlike previous studies, this evaluation examined a homogenous group of providers (all physicians) and controlled for the potential confounding effect of transport destination. However, this study was a retrospective analysis, and baseline characteristics of the two comparison populations were significantly different, thus severely limiting the strength of its conclusions. The authors attempted to adjust for these differences by using standardized mortality ratios and multivariate regressions, but the results still must be interpreted with caution. As all of the HEMS responses are staffed exclusively with physicians, the results may not be generalizable to EMS systems with other staffing configurations. Despite these limitations, this analysis still appears to lend support to an outcome benefit of HEMS transport in a physician-staffed EMS system caring for a severely injured trauma cohort. Interestingly, a recent Cochrane analysis failed to provide any definitive conclusions about staffing models for HEMS [41]. It is presumable that decreasing time to definitive and advanced care should increase patient survival; however, more research involving homogenous models, while also addressing societal cost, appears necessary for the HEMS discussion.

## Evaluative strategies in traumatic injury

Uncontrolled hemorrhage is one of the leading causes of death in trauma patients. As such, early detection and intervention of patients with hypovolemic shock are paramount in trauma resuscitation. The American College of Surgeons Advanced Trauma Life Support (ATLS) course defines four categories of hypovolemic shock on the basis of vital signs with a goal of early identification and intervention for hypovolemic shock. However, this classification scheme has been repeatedly called into question and may not fulfill its intended purpose of adequately predicting outcomes for trauma patients [42-44]. In a pair of articles published in 2013, Mutschler and colleagues describe approaches for predicting clinical course in trauma patients by using BD [45] and the SI for trauma [46].

### The use of base deficit in the assessment of trauma patients

Abnormal BD has been previously associated with increased transfusion requirements [47] and mortality [48], and improvement has been suggested as an indicator of adequate resuscitative efforts. Mutschler and colleagues [45] attempted to validate a previously described BD-based shock classification system [47] by using a retrospective analysis of prospectively collected trauma registry data. In total, 16,305 cases were examined, and 92% of them involved blunt trauma.

A previously derived shock scale based on BD [47] defines class I as BD of less than 2 mmol/L (no shock), class II as BD of 2 to 6 mmol/L (mild shock), class III as BD of 6 to 10 mmol/L (moderate shock), and class IV as BD of more than 10 mmol/L (severe shock). Whereas patients in BD class IV had high rates of hypotension, no group demonstrated significant tachycardia. Furthermore, compared with the ATLS classification system, the BD classification system more accurately predicted mortality (ATLS 31% versus BD 51.5%, *P* <0.001). In their analysis, the authors noted a correlation between worsening BD category and increased injury severity (ISS 19.1, 24.0, 29.5, and 36.7, respectively, for BD of 1 to 4) and mortality (7.4%, 12.4%, 23.9%, and 51.5%, respectively).

The classification system validated by Mutschler and colleagues may have some utility in terms of predicting outcomes in trauma but has several limitations. The study was a retrospective analysis of a cohort with over 92% blunt trauma. Although this raises concerns about the external validity of the classification scheme, the findings by Mutschler and colleagues complement other recent studies demonstrating the utility of BD to predict mortality in blunt trauma [49,50]. In addition, these findings are in line with previous literature demonstrating the inadequacy of vital signs, when used in isolation, for predicting outcomes in potential hypovolemic shock cases [43]. Unfortunately, there is no gold standard for comparison of this BD-based classification system. What is clear, however, is that no single marker can ultimately predict outcomes in patients with shock. However, this study by Mutschler and colleagues adds another inexpensive, readily available tool to diagnostic algorithms for trauma resuscitation.

### The utility of the shock index in the initial evaluation and management of trauma

Although BD shows promise as a tool for predicting hemorrhagic shock in trauma patients, not all facilities have the ability to rapidly obtain this laboratory evaluation in the early phases of resuscitation. In their previously discussed work on BD, Mutschler and colleagues noted a correlation between BD and an increasing SI, defined as the ratio of heart rate to systolic blood pressure [45]. Likewise, previous work has demonstrated the utility of the SI to identify hemodynamic instability [51,52]. The authors therefore performed an analysis of the same registry data as the BD study in an attempt to characterize four groups of patients on the basis of transfusion requirements and outcomes and, in turn, compare this classification scheme with their previously described BD-based system [46].

This study used the same trauma registry data as previously described [45] and examined a total of 21,853 patients. The authors identified four classes of shock based on SI, defined as class I (SI of less than 0.6, no shock), class II (SI of 0.6 to 1, mild shock), class III (SI of 1 to 1.4, moderate shock), and class IV (SI of more than 1.4, severe shock). The results showed that the higher SI classes were, in fact, associated with increased transfusion requirements, with mortality increased from 10.9% in class I to 39.8% in class IV. The authors noted a similar predictive ability for transfusion between the SI-based and the BD-based classification schemes, with AUCs of 0.719 (0.710 to 0.728) and 0.711 (0.703 to 0.720), respectively. Clinically meaningful differences were most often seen between class II and class III, suggesting a cut point SI of 1.0 as a marker of a sicker cohort. This finding is consistent with previous investigations of a valid cut point for mortality using the SI [53]. However, the SI scheme in the current analysis was not directly compared with the BD scheme in terms of outcome measures.

Though not yet validated in a multi-center or prospective study, this classification scheme proposed by Mutschler and colleagues appears to be a readily available and easily usable system to predict the need for transfusion and ultimate mortality in trauma patients. Although it compared favorably to the previously discussed BD classification scheme regarding transfusion requirements, the two schemes were not directly compared in terms of outcomes. Finally, initial SI does seem to be an important predictor of outcomes, but given other studies, it may be the change in SI that has the most predictive ability [52]. Although this study may not be applicable to all systems, it still proposes a simple classification scheme using readily available data that may have clinical utility and certainly warrants further investigation and prospective validation.

## A novel evaluative strategy for smoke inhalation injuries

In patients with burns, SII is a major contributor to morbidity [54]. The primary method for diagnosing SII is bronchoscopy, in which the grade of injury is subjectively determined [55]. In a novel approach, Yamamura and colleagues [56] sought to identify an objective scoring method by using CT scans of the chest to classify the severity and complications of SII. The authors examined 37 patients sequentially presenting with SII to a Japanese hospital, all of whom received serial CT imaging of the chest. Among the studied patients, 68% required endotracheal intubation and the average total body surface area (TBSA) burn was 12%. CT scans were evaluated for bronchial wall thickness (BWT) and compared with those of healthy controls. All patients received bronchoscopy, and degree of bronchial injury was scored by using previously described criteria [57]. Although admission BWT value did not correlate with ultimate outcome (*P* = 0.11), a cutoff of BWT of more than 3.0 mm was found to predict the development of pneumonia with a sensitivity of 79% and a specificity of 96% after receiver operator curve analysis. This compared favorably to bronchoscopy, and respective predictive values were 50% and 70%. Similar predictive results were described for total ventilator days and ICU days.

Although this study does identify a promising new diagnostic algorithm for the identification of SII impacting clinically relevant patient outcomes, it has several limitations. First, patients in this study received more IV fluid than would have been expected on the basis of standard resuscitation algorithms, possibly a confounding variable. Additionally, the sample size was small and included only patients with less than 20% TBSA burns, limiting its generalizability. Finally, perhaps because of the small cohort size, the study failed to demonstrate utility in terms of identifying long-term outcomes or mortality. Thus, although this article provided a promising new use of existing technology and is compelling enough for further investigation, it does not provide sufficient evidence to change current practice patterns.

## The use of biomarkers in the initial evaluation of emergency patients

### Plasma thioredoxin may predict outcomes in post-cardiac arrest patients

The pathophysiology of post-cardiac arrest syndrome involves three distinct processes: brain injury, myocardial dysfunction, and systemic ischemia-reperfusion [58]. This ischemia-reperfusion results in systemic inflammatory and oxidative injury. Timely identification leading to early and aggressive intervention presumably leads to improved outcomes [58]. Indicators of systemic inflammation and neuronal injury, including C-reactive protein, S-100b, and procalcitonin, have been posited as potential markers for disease severity following cardiac arrest, but results have been mixed [59,60]. Mongardon and colleagues [61] recently published an article proposing the use of thioredoxin (TRX), an oxygen scavenger and inflammatory modulator, as a potentially useful marker of disease severity following cardiac arrest.

The authors retrospectively evaluated banked blood samples from 176 adults (over age 18) who had ROSC after cardiac arrest and who were admitted to the medical ICU. TRX levels drawn on admission and on day 1 were able to effectively discriminate survivors from non-survivors, and admission TRX levels were 22 and 72.4 ng/mL (*P* <0.001) in survivors and non-survivors, respectively. However, after the first day of hospitalization, this discrimination was lost. Patients with a cardiac arrest due to VF/VT demonstrated the lowest TRX levels, followed by non-VF/VT cardiac arrest due to cardiac etiologies, followed by those with non-cardiac etiologies. Procalcitonin levels were also examined and worked well for discriminating survival, including early and late death, when measured at admission through day 3. However, procalcitonin was not directly compared with TRX. Overall mortality in this cohort was 61%; 74% experienced post-resuscitation shock, whereas 89% of patients had been treated with induced therapeutic hypothermia.

According to these results published by Mongardon and colleagues [61], TRX may have some benefit in terms of identifying patients at high mortality from cardiac arrest following resuscitation if analyzed early in the clinical course. However, a direct comparison of TRX with previously validated biochemical markers has not been performed. Although definitive conclusions cannot be drawn from this single study, the utility and cost-effectiveness of TRX in identifying patients at risk of developing post-cardiac arrest syndrome are questionable. Several inexpensive and reliable predictors of poor outcomes following ROSC are already available, and further evaluation of TRX is required prior to adding this assay to any prognostic algorithm.

### The utility of plasma matrix metalloprotease in the diagnosis of aortic dissection

Acute aortic dissection (AAD) is a condition with high morbidity in which timely recognition and management are critical [62]. However, diagnosis in the acute setting can be challenging [63]. Standard approaches for definitive diagnosis include CT angiography or magnetic resonance imaging of the aorta or transesophageal echocardiography [64]. All of these approaches require substantial resources and may not be available in all settings. Serum biomarkers have been proposed as a potential diagnostic modality, and D-dimer testing has shown high sensitivity but low specificity for the diagnosis of AAD in the emergency department [65-67]. Although D-dimer may have some limited benefit in ruling out AAD for very low-risk patients, it has less utility in the definitive diagnosis of AAD [68].

Matrix metalloproteinases (MMPs) are key molecular modulators of large-vessel disease and have been posited as a key mediator of aortic dissection [69]. Giachino and colleagues [70] evaluated MMPs for the diagnosis of AAD in the acute setting. The group examined blood samples obtained from patients suspected of having AAD for MMPs and several other biomarkers, including D-dimer. All patients were subsequently evaluated with CT angiography of the chest and abdomen. Of 126 patients evaluated over the 26-month study period, 53 patients (41.3%) were ultimately diagnosed with AAD. MMP8 and MMP9 levels were significantly elevated in patients diagnosed with AAD (36.4 versus 13.2 ng/mL, *P* <0.0001 and 169.2 versus 80.5 ng/mL, *P* = 0.0001, respectively). D-dimer levels were also significantly elevated in patients ultimately diagnosed with AAD as compared with those without AAD (7.16 versus 1.34 μg/mL, *P* <0.0001). Both MMP assays correlated significantly with D-dimer levels (r = 0.32 and r = 0.27, respectively). Sensitivity and specificity were 100% and 9.5% for MMP8 and 96.2% and 16.2% for MMP9. D-dimer demonstrated sensitivity and specificity of 97.6% and 32.8% in this cohort. When MMP8 was added to D-dimer, sensitivity and specificity were 100% and 16.4%, respectively, with an AUC of 0.87 (*P* = 0.034).

The diagnosis of AAD is challenging in the acute setting, and this prospective evaluation of the novel use of biomarkers for the rapid identification of AAD adds a promising tool to diagnostic algorithms. However, MMP evaluation added only marginal benefit to the readily available D-dimer. As such, use of MMPs cannot be recommended at this time in the diagnosis of AAD. However, the high sensitivity of these assays may prove useful in rule-out algorithms, particularly when used in combination with D-dimer.

## Conclusions

Several articles published in 2013 focused on the management of cardiac arrest, traumatic injury, and other emergency conditions. With regard to medication administration during cardiac arrest, a large meta-analysis of anti-arrhythmics found no benefit in survival to discharge for any anti-arrhythmic [10]. Likewise, the utility of epinephrine was again questioned [21]. Though limited by study design, these articles highlight the need for rigorous controlled studies defining targeted populations that might benefit from such therapies. In regard to TOR for OHCA, currently used TOR rules [25] may prematurely terminate efforts in patients with the potential for meaningful recovery. Further investigation must be conducted to better determine the specific factors associated with survivability after OHCA.

The issue of HEMS transport for critically ill and injured patients has a long history [36], and further analysis has again suggested a survival advantage to HEMS transport [41], despite issues with study design and generalizability. Novel evaluative strategies for trauma patients, including the use of BD [45] and SI [46], have been suggested, although any additional benefit over currently used instruments is questionable. Likewise, the use of TRX to identify patients with potentially favorable outcomes after cardiac arrest [61] and the use of MMPs to rule out AAD [70] are interesting, but practicality and cost-effectiveness are uncertain and require further investigation. Overall, articles published in 2013 pertaining to the management of trauma and OHCA raised more questions than they answered but provided ample opportunity for further scientific inquiry.

## **Note:**

 This article is part of a collection of Year-in-review articles in *Critical Care*. Other articles in this series can be found at http://ccforum.com/series/Yearinreview2013.

## Abbreviations

AAD: acute aortic dissection
ALS: advanced life support
ATLS: advanced trauma life support
AUC: area under receiver operator curve
BD: base deficit
BWT: bronchial wall thickness
CI: confidence interval
CPC: cerebral performance category
CPR: cardiopulmonary resuscitation
CT: computerized tomography
EMS: emergency medical services
GEMS: ground emergency medical services
HEMS: helicopter emergency medical services
ISS: injury severity score
IV: intravenous
MMP: matrix metalloproteinase
OHCA: out-of-hospital cardiac arrest
OR: odds ratio
ROSC: return of spontaneous circulation
RR: risk ratio
SI: shock index
SII: smoke inhalation injury
TBSA: total body surface area
TOR: termination of resuscitation
TRX: thioredoxin
VF: ventricular fibrillation
VT: ventricular tachycardia
